# Association of Treatment Advances With Survival Rates in Pediatric Patients With Nasopharyngeal Carcinoma in China, 1989-2020

**DOI:** 10.1001/jamanetworkopen.2022.0173

**Published:** 2022-03-02

**Authors:** Yu-Jing Liang, Li-Ting Liu, Yang Li, Pan Wang, Mei-Juan Luo, Dong-Xiang Wen, Qiu-Yan Chen, Hai-Qiang Mai

**Affiliations:** 1 Sun Yat-sen University Cancer Center, State Key Laboratory of Oncology in South China; 2 Department of Nasopharyngeal Carcinoma, Sun Yat-sen University Cancer Center, Guangzhou, China; 3 Collaborative Innovation Center for Cancer Medicine; Guangdong Key Laboratory of Nasopharyngeal Carcinoma Diagnosis and Therapy, Guangzhou, China; 4 Department of Radiotherapy, Jiangsu Cancer Hospital, Jiangsu Institute of Cancer Research, Affiliated Cancer Hospital of Nanjing Medical University, Nanjing, China

## Abstract

**Importance:**

Advanced techniques and treatment methods have been found to be associated with improved survival rates in adults with nasopharyngeal carcinoma (NPC); however, not much is known about associations in pediatric patients.

**Objective:**

To investigate whether advanced imaging diagnosis, radiotherapy (RT) technology, and treatment modality are associated with survival in pediatric patients with NPC.

**Design, Setting, and Participants:**

In this retrospective cohort study, 810 pediatric patients ages 21 years and younger with nonmetastatic NPC diagnosed from 1989 to 2020 at a single cancer center in China were included. Data were analyzed from April through December 2021.

**Exposures:**

Patients were divided into 3 groups by initial treatment date (ie, 1989-2002, 2003-2011, and 2012-2020). Associations between advances in technology and treatment and survival were investigated. Comparisons of advances vs older technology and treatments included those in imaging diagnosis (magnetic resonance imaging [MRI] vs computed tomography [CT] and positron emission tomography [PET]–CT with MRI vs CT), radiotherapy (RT) techniques (intensity-modulated RT [IMRT] or TomoTherapy [TOMO] vs 2-dimensional conventional radiotherapy [2D-CRT] or 3-dimensional conventional radiotherapy [3D-CRT]), and treatment methods (concurrent chemoradiotherapy [CCRT] vs RT alone, induction chemotherapy [IC] with CCRT vs RT alone, and CCRT or RT with adjuvant chemotherapy [AC] vs RT alone).

**Main Outcomes and Measures:**

The primary end point was progression-free survival (PFS). Secondary end points were overall survival (OS), distant metastasis–free survival, and locoregional recurrence–free survival. Cox and competing-risks regression were used to estimate hazard ratios (HRs) and 95% CIs for associations between variables and survival.

**Results:**

Among 810 pediatric patients with NPC, the median (IQR) age was 18 (15-20) years, and there were 577 [71.2%] male patients. This included 122 patients in the 1989 to 2002 period, 212 patients in the 2003 to 2011 period, and 476 patients in the 2012 to 2020 period. The 5-year PFS and OS rates increased, respectively, from 65.9% (95% CI, 56.6%-73.7%) and 69.9% (95% CI, 60.7%-77.4%) in 1989 to 2002 to 79.8% (95% CI, 73.7%-84.7%) and 86.2% (95% CI, 80.6%-90.3%) in 2003 to 2011, then 88.1% (95% CI, 84.2%-91.1%) and 95.0% (95% CI, 91.5%-97.0%) in 2012 to 2020. The 5- year cumulative incidence of distant metastasis rate was similar in the 3 periods (1989-2002: 11.7% [95% CI, 7.0%- 19.4%]; 2003-2011: 18.0% [95% CI, 13.4%-24.0%]; 2012-2020: 10.4% [95% CI, 7.6%-14.1%], while the 5-year cumulative incidence of locoregional recurrence rate decreased from 22.5% (95% CI, 15.9%-31.3%) in the first period to 2.9% (95% CI, 1.3%-6.3%) in the second period, remaining stable in the third period, at 4.3% (95% CI, 2.4%-7.6%). Advances in imaging diagnosis (MRI vs CT: hazard ratio [HR], 0.25 [95% CI, 0.17-0.38]; PET-CT with MRI vs CT: HR, 0.41 [95% CI, 0.27-0.62]), radiotherapy techniques (IMRT or TOMO vs 2D-CRT or 3D-CRT: HR, 0.42 [95% CI, 0.30-0.59]), and treatment methods (CCRT vs RT alone: HR, 0.55 [95% CI, 0.32-0.96]; IC with CCRT vs RT alone: HR, 0.59 [95% CI, 0.38-0.91]; CCRT or RT with AC vs RT alone: HR, 0.48 [95% CI, 0.25-0.91]) were associated with improved PFS.

**Conclusions and Relevance:**

This study found that advanced techniques and treatment methods were associated with improved survival rates in pediatric patients with NPC, but distant failure remained a key challenge.

## Key Points

**Question:**

Are advances in technology associated with survival rates in pediatric patients with nasopharyngeal carcinoma (NPC)?

**Findings:**

In this cohort study of 810 pediatric patients in China with NPC, advanced techniques and treatment methods were associated with improvements in progression-free survival rates, but distant failure remained a key challenge.

**Meaning:**

This study found that advanced technology and treatment were associated with improved survival rates in pediatric patients with NPC, but distant failure remained a key challenge.

## Teaser

This cohort study investigates the association of advances in imaging diagnosis, radiotherapy techniques, and treatment methods with survival among pediatric patients with nasopharyngeal carcinoma in China from 1989 to 2020.

## Introduction

Nasopharyngeal carcinoma (NPC) is considered a rare type of malignant neoplasm globally, but it is endemic in Southeastern Asia and Eastern Asia, especially in some provinces in southeastern China, with a peak incidence in individuals aged 40 to 60 years.^1^ However, a subpeak is also found among individuals between the ages of 10 and 20 years.^2^ Owing to the hidden location of NPC, the nonspecific nature of nasal and auditory symptoms, and the difficulty of clinical examination of the nasopharynx, most individuals have locoregionally advanced stages at diagnosis.^3^ Compared with adults, pediatric patients with NPC seem more likely to have advanced stages (ie, stages III and IV) with more lymph node metastasis.^4^ In endemic areas, most pathological cases in pediatric patients are nonkeratinizing subtypes, which are always associated with Epstein-Barr virus (EBV) infection. External radiotherapy (RT) is the primary curative treatment method, and with RT alone, the 10-year disease-specific survival rate among pediatric patients with NPC patients was reported to be 98% with stage I disease but 60% with stage II disease.^5^

Considerable advances in technology, including in image diagnosis, RT techniques, and treatment modalities of RT plus sequential chemotherapy, have been reported to be associated with improved survival rates in pediatric patients with NPC, with 5-year overall survival (OS) rates between 80% and 95%.^4,6^ Regarding image diagnosis techniques, compared with conventional computed tomography (CT), magnetic resonance imaging (MRI) provides better resolution and visualization of soft tissues, as well as a lack of radiation, which have been associated with a more accurate diagnosis of clinical stage of NPC.^7^ Moreover, the application of intensity-modulated RT (IMRT) and multimodal treatment combining RT and cisplatin-based chemotherapy are associated with improved survival.^8,9,10,11^ For a long time, diagnosis and treatment for pediatric patients with NPC usually followed clinical practice guidelines for adults, despite potential variations in pathogenesis and progression. Although recommendations have been recently published to improve diagnosis and treatment homogeneity in this special population,^12^ little is known about the association of advances in technology and treatment with survival rates in pediatric patients with NPC. In addition, as a promising marker for adult patients,^13,14^ the role of pretreatment EBV DNA and its optimal cutoff value have not been thoroughly investigated in pediatric patients with NPC, to our knowledge. Therefore, we carried out a large-scale, long-term–follow-up cohort study in pediatric patients with nonmetastatic NPC who were diagnosed and treated between 1989 and 2020. We aimed to explore associations between advances in techniques and treatment and survival rates in pediatric patients with NPC by comparing survival rates over the past few decades.

## Methods

This cohort study followed Strengthening the Reporting of Observational Studies in Epidemiology (STROBE) reporting guideline and was approved by the Institutional Review Committee and Ethics Committee of the Sun Yat-sen University Cancer Center (SYSUCC). Patient data remained anonymous before and during analysis; additionally, owing to the observational nature of the study, the requirement for informed consent was waived by SYSUCC.

### Patients

Using the NPC-specific database within the big data intelligence framework Yidu Cloud (Yidu Cloud Technology Company) and the prospectively created database at SYSUCC in China, we screened medical records of 835 pediatric patients diagnosed with nonmetastatic NPC between November 1989 and November 2020. Eligible patients were reclassified based on clinical and imaging data according to the Union for International Cancer Control (UICC) *TNM Classification of Malignant Tumours*, 8th edition.^15^ The inclusion criteria were (1) pathology-demonstrated NPC, (2) age 21 years or younger, (3) untreated at first diagnosis, (4) receiving RT or chemotherapy previously, (4) without distant metastasis, and (5) adequate hematological function (ie, white blood cell count ≥ 3000/μL [≥3 × 10^9^/L]; platelet count ≥ 100 × 10^3^/μL [≥100 × 109/L], and hemoglobin ≥ 9 g/dL [90 g/L]), hepatic function (ie, serum bilirubin ≤2.0 × the upper limit of normal [ULN] and alanine aminotransferase and aspartate aminotransferase ≤2.5 × ULN), and kidney function (ie, creatinine ≤1.5 × ULN). Patients with previous malignant neoplasms or female patients who were pregnant or breastfeeding before or during treatment were excluded. Ultimately, 25 patients were excluded and 810 patients were identified and included (eFigure 1 in the Supplement).

### Diagnosis and Treatment

Before treatment, each patient was assessed by medical history, physical examination, nasopharyngeal endoscopy and biopsy for pathology, MRI or CT of the nasopharynx and neck, and blood tests. Before RT, dental evaluation and prophylaxis or treatment were highly recommended. To detect distant metastasis, chest x-ray or CT, abdominal ultrasound or CT, and skeletal scintigraphy or PET-CT were applied.

All patients received radical RT with 2-dimensional or 3-dimensional conventional RT (2D-CRT or 3D-CRT), IMRT, or TomoTherapy. The plan design, radiation dosage, and implementation were based on a previous study.^16^ Chemotherapy was administered intravenously every 3 weeks, with common regimens of induction chemotherapy (IC) and adjuvant chemotherapy (AC), including the docetaxel, cisplatin, and fluorouracil (TPF) combination: docetaxel (60-75 mg/m^2^) and cisplatin (60-75 mg/m^2^) in combination with 5-fluorouracil (3-3.75 g/m^2^ continuous intravenous for 120 hours); the cisplatin and fluorouracil (PF) combination: cisplatin (80 mg/m^2^) plus 5-fluorouracil (4 g/m^2^ continuous intravenous for 120 hours); and the docetaxel with cisplatin (TP) combination: docetaxel (75 ~ 80 mg/m^2^) and cisplatin (80 mg/m^2^). Concurrent chemotherapy comprised a regimen of mainly cisplatin or nedaplatin, with a dosage of 80 or 100 mg/m^2^.

### Clinical Outcome and Follow-up

The primary end point was progression-free survival (PFS), which was defined as the time from pathology diagnosis to documented progression or death from any cause. The secondary end points were OS, distant metastasis–free survival, and locoregional recurrence–free survival. The definition of OS was the time of pathology diagnosis to death from any cause. Distant metastasis–free survival and locoregional recurrence–free survival were defined as the time from pathology diagnosis to documented distant metastasis or recurrence, respectively. The end of the follow-up period for outcome ascertainment was April 1, 2021. After treatment, follow-up inspections were recommended every 3 months for the first 3 years and every 6 months thereafter until death.

### Statistical Analysis

A previous study from our institute reported that all patients received 2D-CRT before 2002 and that EBV DNA detection was carried out mainly after 2003. Moreover, IMRT became the main RT technique after 2012.^17,18^ Therefore, patients included in this study were divided into 3 groups according to their initial treatment dates, with 2002 to 2003 and 2011 to 2012 as the annual cutoff points. Clinical characteristics were demonstrated as median (IQR) or number (percentage) and analyzed using χ^2^ test or Fisher exact test. Cutoff values for continuous variables were chosen based on routine clinical applications.^19^ The new bioinformatics tool X-Tile version 3.6.1 (Yale School of Medicine) was used to obtain the exploratory optimal cut point.^20^ Kaplan-Meier survival curves were used to calculate survival rates, which were compared with the log-rank test.

In our study, 210 missing values were identified for EBV DNA, 47 missing values for viral capsid antibody (VCA-IgA), 47 missing values for early antigen antibody (EA-IgA), and 62 missing values for body mass index (BMI; calculated as weight in kilograms divided by height in meters squared). Cox analyses were used to estimate hazard ratios (HRs) and 95% CIs for associations between variables and survival. Competing-risks regression and cumulative incidence of events using Fine and Gray method were applied to compare distributions of time with locoregional recurrence and distant metastasis events, accounting for deaths without experiencing failure. Multiple imputation was performed to handle missing values as a sensitivity analysis. Univariable analysis was first performed for potential prognostic variables, including performance status score (possible score range, 70-80 or 90-100, evaluated via Karnofsky Performance Scale or Lansky Play-Performance Scale), BMI, sex, age, image technique, T stage, N stage, treatment, RT technique, calendar period, VCA-IgA level, EA-IgA level, and EBV DNA level, and variables with *P* value < .05 were selected into the final multivariable Cox analysis. Statistical analyses were performed using R statistical software version 4.0.2 (R Project for Statistical Computing) and Stata statistical software version 15.0 (StataCorp). All tests were conducted at the significance level of α = .05 (2-tailed).

## Results

### Patient Characteristics

This cohort comprised 810 consecutive pediatric patients with nonmetastatic (UICC 8th edition, stages I-Va) NPC (577 [71.2%] male patients and 233 [28.8%] female patients) aged 2 to 21 years (median [IQR] age, 18 [15-20] years). This included 122 patients in the 1989 to 2002 period, 212 patients in the 2003 to 2011 period, and 476 patients in the 2012 to 2020 period. Detailed baseline data, including BMI, performance status, sex, age, family history of NPC, family history of cancer, clinical stage, pathologic type, image and RT technique, and treatment or pretreatment EBV DNA level across periods, are displayed in Table 1.

**Table 1.** Baseline Patient Characteristics

Characteristic	Patients, No. (%)^a^				*P* value^b^
	Total (N = 810)	1989-2002 (n= 122)	2003-2011 (n = 212)	2012-2020 (n = 476)	
BMI, mean (SD)	19.6 (4.2)	18.3 (3.0)	19.6 (4.3)	19.9 (4.4)	.002^c^
PS^d^					
90-100	705 (87.0)	116 (95.1)	155 (73.1)	434 (91.2)	<.001
70-80	105 (13.0)	6 (4.9)	57 (26.9)	42 (8.8	
Sex					
Female	233 (28.8)	35 (28.7)	63 (29.7)	135 (28.4)	.94
Male	577 (71.2)	87 (71.3)	149 (70.3)	341 (71.6)	
Age, y					
Median (IQR)	18 (15-20)	18 (15-20)	18 (16-20)	18 (15-20)	NA
≤18	463 (57.2)	66 (54.1)	110 (51.9)	287 (60.3)	.09
>18	347 (42.8)	56 (45.9)	102 (48.1)	189 (39.7)	
Family history					
NPC					
No	772 (95.3)	114 (93.4)	201 (94.8)	457 (96.0)	.45
Yes	38 (4.7)	8 (6.6)	11 (5.2)	19 (4.0)	
Cancer					
No	699 (86.3)	102 (83.6)	182 (85.8)	415 (87.2)	.58
Yes	111 (13.7)	20 (16.4)	30 (14.2)	61 (12.8)	
T stage					
1	20 (2.5)	5 (4.1)	7 (3.3)	8 (1.7)	<.001
2	78 (9.6)	21 (17.2)	22 (10.4)	35 (7.4)	
3	397 (49)	67 (54.9)	107 (50.5)	223 (46.8)	
4	315 (38.9)	29 (23.8)	76 (35.8)	210 (44.1)	
N stage					
0	53 (6.5)	18 (14.8)	20 (9.4)	15 (3.2)	<.001
1	220 (27.2)	40 (32.8)	72 (34)	108 (22.7)	
2	360 (44.4)	51 (41.8)	91 (42.9)	218 (45.8)	
3	177 (21.9)	13 (10.7)	29 (13.7)	135 (28.4)	
Overall stage					
I	9 (1.1)	2 (1.6)	4 (1.9)	3 (0.6)	<.001
II	40 (4.9)	12 (9.8)	10 (4.7)	18 (3.8)	
III	334 (41.2)	68 (55.7)	100 (47.2)	166 (34.9)	
IVa	427 (52.7)	40 (32.8)	98 (46.2)	289 (60.7)	
Pathologic type					
WHO II	5 (0.6)	1 (0.8)	4 (1.9)	0	.009^e^
WHO III	805 (99.4)	121 (99.2)	208 (98.1)	476 (100)	
Image technique					
CT	184 (22.7)	122 (100)	62 (29.2)	0	<.001
MRI	350 (43.2)	0	131 (61.8)	219 (46)	
PET-CT with MRI	276 (34.1)	0	19 (9.0)	257 (54.0)	
VCA-IgA					
Median (IQR)	80 (40-320)	160 (80-320)	160 (60-320)	80 (20-160)	NA
<80	312 (38.5)	36 (29.5)	55 (25.9)	221 (46.4)	<.001
≥80	498 (61.5)	86 (70.5)	157 (74.1)	255 (53.6)	
EA-IgA					
Median (IQR)	10 (0-40)	5 (0-20)	10 (0-40)	10 (0-40)	NA
<10	338 (41.7)	75 (61.5)	86 (40.6)	177 (37.2)	<.001
≥10	472 (58.3)	47 (38.5)	126 (59.4)	299 (62.8)	
EBV DNA, copies/mL^f^					
Median (IQR)	1390 (0-15100)	NA	218.7 (0-20 700)	1740 (85.3-13 700)	NA
<4000	364 (60.7)	NA	81 (61.8)	283 (60.3)	.84^e^
≥4000	236 (39.3)	NA	50 (38.2)	186 (39.7)	
Treatment					
RT alone	112 (13.8)	75 (61.5)	22 (10.4)	15 (3.2)	<.001
IC with RT	111 (13.7)	27 (22.1)	45 (21.2)	39 (8.2)	
CCRT	126 (15.6)	8 (6.6)	60 (28.3)	58 (12.2)	
IC with CCRT	337 (41.6)	12 (9.8)	81 (38.2)	244 (51.3)	
CCRT or RT with AC	124 (15.3)	0	4 (1.9)	120 (25.2)	
RT technique					
2D-CRT	220 (27.2)	116 (95.1)	101 (47.6)	3 (0.6)	<.001
3D-CRT	1 (0.1)	0 (0.0)	1 (0.5)	0 (0.0)	
IMRT	576 (71.1)	6 (4.9)	110 (51.9)	460 (96.6)	
TOMO	13 (1.6)	0 (0.0)	0 (0.0)	13 (2.7)	

Abbreviations: 2D-CRT, 2-dimensional conventional radiotherapy; 3D-CRT, 3-dimensional conventional radiotherapy; AC, adjuvant chemotherapy; BMI, body mass index (calculated as weight in kilograms divided by height in meters squared); CCRT, concurrent chemoradiotherapy; CT, computed tomography; EA, early antigen; EBV, Epstein-Barr virus; IC, induction chemotherapy; IgA, immunoglobulin; IMRT, intensity modulated radiation therapy; MRI, magnetic resonance imaging; NA, not applicable; NPC, nasopharyngeal carcinoma; PET, positron emission computer tomography; PS, performance-status score; RT, radiotherapy; TOMO, TomoTherapy; VCA, viral capsid antigen; WHO, World Health Organization.

^a^ There were 62 missing values for BMI (7.7%), 47 missing values for VAC-IgA (5.8%), 47 missing values for EA-IgA (5.8%), and 210 missing values for EBV DNA (25.9%).

^b^ *P* value calculated with unadjusted χ^2^ test unless otherwise stated.

^c^ *P* value calculated with analysis of variance.

^d^ Evaluated by Karnofsky Performance Scale or Lansky Play-Performance Scale.

^e^ *P* value calculated with Fisher exact test.

^f^ EBV DNA was not available in 1989 to 2002 because this test was carried out mainly after 2003.

The proportion of patients in advanced stages (ie, stages III-IVa) increased from 108 patients (88.5%) in 1989 to 2002 to 198 patients (93.4%) in 2003 to 2011. All patients completed RT. The proportion of patients receiving 2D-CRT decreased, from 116 patients in 1989 to 2002 (95.1%) to 101 patients in 2003 to 2011 (47.6%) and 3 patients in 2012 to 2020 (0.6%). The overall median (IQR) RT doses were 70 (68-72) Gy in the nasopharynx and 60 (60-64) GY in involved areas of the neck for 30 to 35 fractions. Associated with the development of the technique, IMRT replaced 2D-CRT and 3D-CRT as the main RT method, with 6 patients (4.9%) in 1989 to 2002, 110 patients (51.9%) in 2003 to 2011, and 460 patients (96.6%) in 2012 to 2020 receiving IMRT. The overall median (IQR) RT doses were 70 (68-70) Gy in the nasopharynx and 66 (62-70) Gy in involved areas of the neck for 30 to 33 fractions in the IMRT era. Before 2003, CT was the only image diagnosis technique, received by all patients. MRI or PET-CT combined with MRI became the main imaging diagnostic staging technique thereafter, with 150 patients (70.8%) in 2003 to 2011 and 476 patients (100%) in 2012 to 2020 receiving the technique (Table 1).

The proportion of patients who received RT alone decreased from 75 individuals (61.5%) in the first period to 15 individuals (3.2%) in the last period, and IC with RT was similar in the first two periods (1989-2002: 27 individuals [22.1%]; 2003-2012: 45 individuals [21.2%]) and decreased in the last period (39 individuals [8.2%]). The proportion of patients receiving concurrent chemoradiotherapy (CCRT) increased from 8 individuals (6.6%) at the beginning to 60 individuals (28.3%) in the second period and then decreased to 58 individuals (12.2%) in the last period. An increasing proportion of patients received IC with CCRT, with 12 patients (9.8%) in 1989 to 2002, 81 patients (38.2%) in 2003 to 2011, and 244 patients (51.3%) in 2012 to 2020 receiving the treatment. The proportion of patients receiving CCRT or RT with AC increased from 0 individuals in 1989 to 2002 to 4 individuals (1.9%) in 2003 to 2011 and 120 individuals (25.2%) in 2012 to 2020.

### Survival

**Figure 1.**
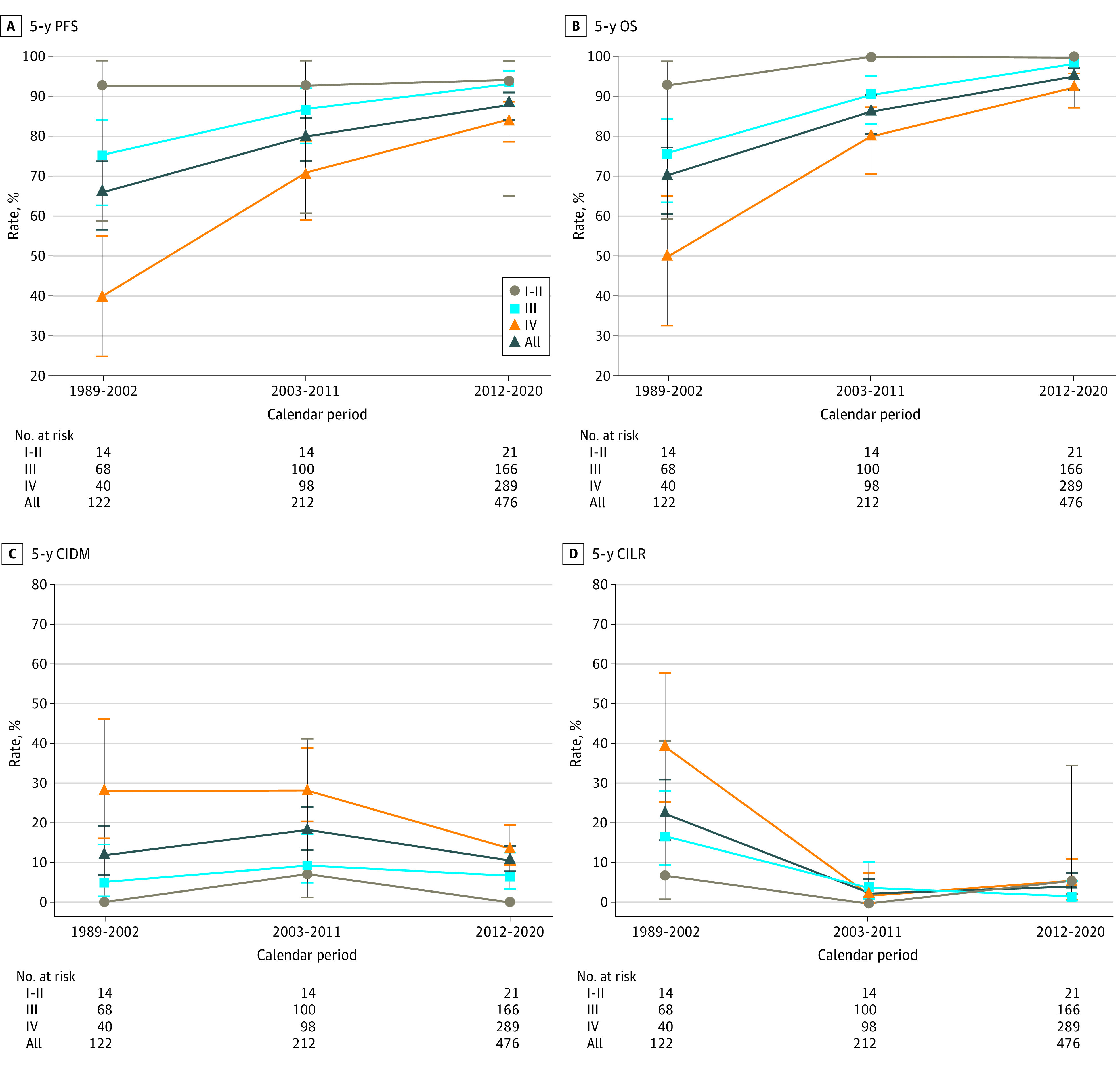
5-Year Survival Rates by Stage and Period The 5-year progression-free survival (PFS), overall survival (OS), cumulative incidence of distant metastasis (CIDM), and cumulative incidence of locoregional recurrence (CILR) rates are presented.

The median (IQR) follow-up time was 56 (24-101) months in the entire cohort. The 5-year PFS, OS, cumulative incidence of distant metastasis (CIDM), and cumulative incidence of locoregional recurrence (CILR) rates for stages I to II, III, and IVa in each period are displayed in Figure 1. In general, the 5-year PFS and OS rates increased in the 3 periods, from 65.9% (95% CI, 56.6%-73.7%) and 69.9% (95% CI, 60.7%-77.4%) in 1989 to 2002 to 79.8% (95% CI, 73.7%-84.7%) and 86.2% (95% CI, 80.6%- 90.3%) in 2003 to 2011 and 88.1% (95% CI, 84.2%-91.1%) and 95.0% (95% CI, 91.5%-97.0%) in 2012 to 2020, respectively. The 5-year CIDM rate was similar in the 3 periods (1989-2002: 11.7% [95% CI, 7.0%-19.4%]; 2003-2011: 18.0% [95% CI, 13.4%-24.0%]; 2012-2020: 10.4% [95% CI, 7.6%-14.1%], while the 5-year CILR rate decreased from 22.5% (95% CI, 15.9%-31.3%) in the first period to 2.9% (95% CI, 1.3%-6.3%) in the second period, remaining stable in the third period, at 4.3% (95% CI, 2.4%-7.6%).

Given that there is less follow-up time with new patients, a sensitivity analysis including 235 patients with mature follow-up time with an initial treatment date of 2012 to 2016 was performed. In this analysis, the 5-year PFS also increased in the 3 periods. In this subgroup of patients, the median (IQR) follow-up time was 64 (47-79). The 5-year PFS for stages I to II, III, and IVa and all patients in each period were 92.9% (95% CI, 59.1%-99.0%), 92.7% (95% CI, 84.5%-96.7%), 80.6% (95% CI, 72.6%-86.5%), and 85.8% (95% CI, 80.5%-89.8%), respectively; the PFS for stages III and IVa and all patients were higher than those in 1989 to 2002 and 2003-2011 (eFigure 2 in the Supplement).

In univariable Cox analysis, the RT technique IMRT and treatment modalities of RT in combination with chemotherapy were associated with better PFS in univariable analyses: IMRT or TomoTherapy vs 2D-CRT or 3D-CRT: HR, 0.42 (95% CI, 0.30-0.59); CCRT vs RT alone: HR, 0.55 (95% CI, 0.32-0.96); IC with CCRT vs RT alone: HR, 0.59 (95% CI, 0.38-0.91); CCRT or RT with AC vs RT alone: HR, 0.48 (95% CI, 0.25-0.91) (eTable 1 in the Supplement). Advances in imaging diagnosis (MRI vs CT: HR, 0.25 [95% CI, 0.17-0.38]; PET-CT with MRI vs CT: HR, 0.41 [95% CI, 0.27-0.62]) were also associated with improved PFS. After adjustments in multivariable Cox analysis, T stage (T4 vs T1-3: HR, 2.39 [95% CI, 1.42-4.03]), N stage (N3 vs N0-1: HR, 2.03 [95% CI, 1.01-4.09]), and EBV DNA level (≥4000 vs <4000 copies/mL: HR, 2.11 [95% CI, 1.25-3.56]) were independent prognostic factors associated with PFS, and similar results were observed for OS (Table 2). Results of analyses using imputed data were consistent with core results of the complete data (eTables 2-4 in the Supplement).

**Table 2.** Multivariable Analyses of Prognostic Factors by Outcome From 2003 to 2020

Factor	HR (95%CI)^a^			
	PFS	OS	CIDM	CILR
BMI	0.95 (0.88-1.02)	0.91 (0.81-1.03)	0.99 (0.93-1.05)	0.90 (0.72-1.13)
Image technique	0.35 (0.11-1.06)	0.16 (0.04-0.60)	0.29 (0.09-0.95)	NA^b^
T stage	2.39 (1.42-4.03)	3.13 (1.37-7.13)	2.51 (1.45-4.35)	2.35 (0.72-7.68)
N stage				
0-1	1 [Reference]	1 [Reference]	1 [Reference]	1 [Reference]
2	1.06 (0.53-2.09)	1.47 (0.54-4.0)	1.07 (0.52-2.19)	1.14 (0.26-5.01)
3	2.03 (1.01-4.09)	1.98 (0.65-6.03)	2.18 (1.04-4.57)	0.99 (0.17-5.91)
Treatment	0.70 (0.20-2.48)	NA^b^	1.06 (0.20-5.57)	0.28 (0.02-4.43)
RT technique	1.45 (0.47-4.45)	2.5 (0.53-11.74)	1.37 (0.43-4.38)	NA^b^
Calendar period				
2003-2011	1 [Reference]	1 [Reference]	1 [Reference]	1 [Reference]
2012-2020	0.72 (0.37-1.40)	0.46 (0.19-1.10)	0.61 (0.30-1.25)	1.48 (0.34-6.53)
EBV DNA level^c^	2.11 (1.25-3.56)	1.85 (0.83-4.15)	1.97 (1.14-3.40)	0.87 (0.30-2.56)

Abbreviations: BMI, body mass index (calculated as weight in kilograms divided by height in meters squared); CIDM, cumulative incidence of distant metastasis; CILR, cumulative incidence of locoregional recurrence; EBV, Epstein-Barr virus; HR, hazard ratio; NA, not applicable; OS, overall survival; PFS, progression-free survival; RT, radiotherapy.

^a^ All HRs were calculated with an adjusted Cox proportional hazard model in PFS and OS and with a subdistribution hazard function model in CIDM and CILR. HRs were calculated for T stage (T4 vs T1-3), N stage (N2 and N3 vs N0-1), image technique (magnetic resonance imaging vs computed tomography), RT technique (intensity modulated radiation therapy or TomoTherapy vs 2-dimensional or 3-dimensional conventional radiotherapy), treatment (RT with chemotherapy vs RT alone), and EBV DNA level (≥4000 vs <4000 copies/ mL).

^b^ Extreme value.

^c^ EBV DNA was not available in 1989 to 2002 because this test was carried out mainly after 2003.

**Figure 2.**
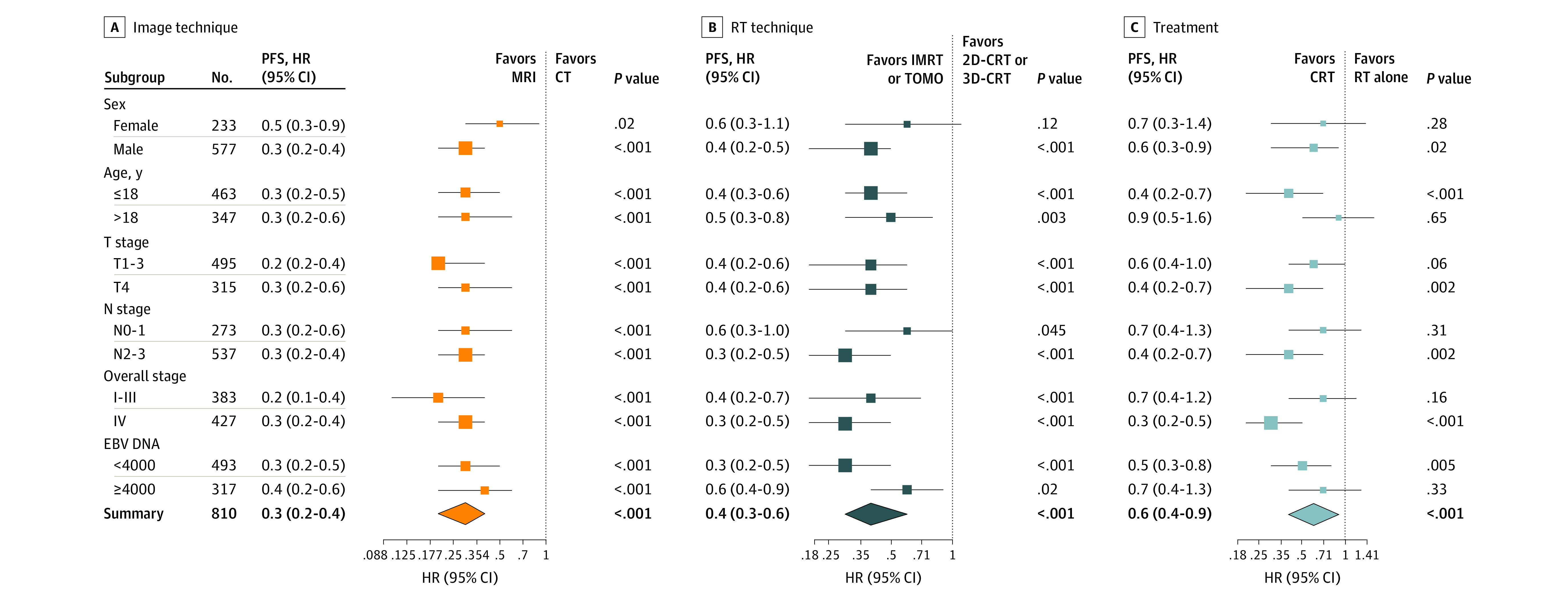
Association of Technique and Treatment Advances With Progression-Free Survival (PFS) by Subgroup 2D-CRT indicates 2-dimensional conventional radiotherapy; 3D-CRT, 3-dimensional conventional radiotherapy; CT, computed tomography; EBV, Epstein-Barr virus; HR, hazard ratio; IMRT, intensity modulated radiation therapy; MRI, magnetic resonance imaging; RT, radiotherapy; TOMO, TomoTherapy.

Subgroup analysis was performed for imaging, RT technique, and chemotherapy. Compared with 2D-CRT or 3D-CRT and RT alone, IMRT or TomoTherapy and RT combined with chemotherapy were factors associated with protection for all subgroups and were statistically significant in most subgroups. MRI as an image diagnosis technique was a variable associated with protection for all subgroups (Figure 2).

**Figure 3.**
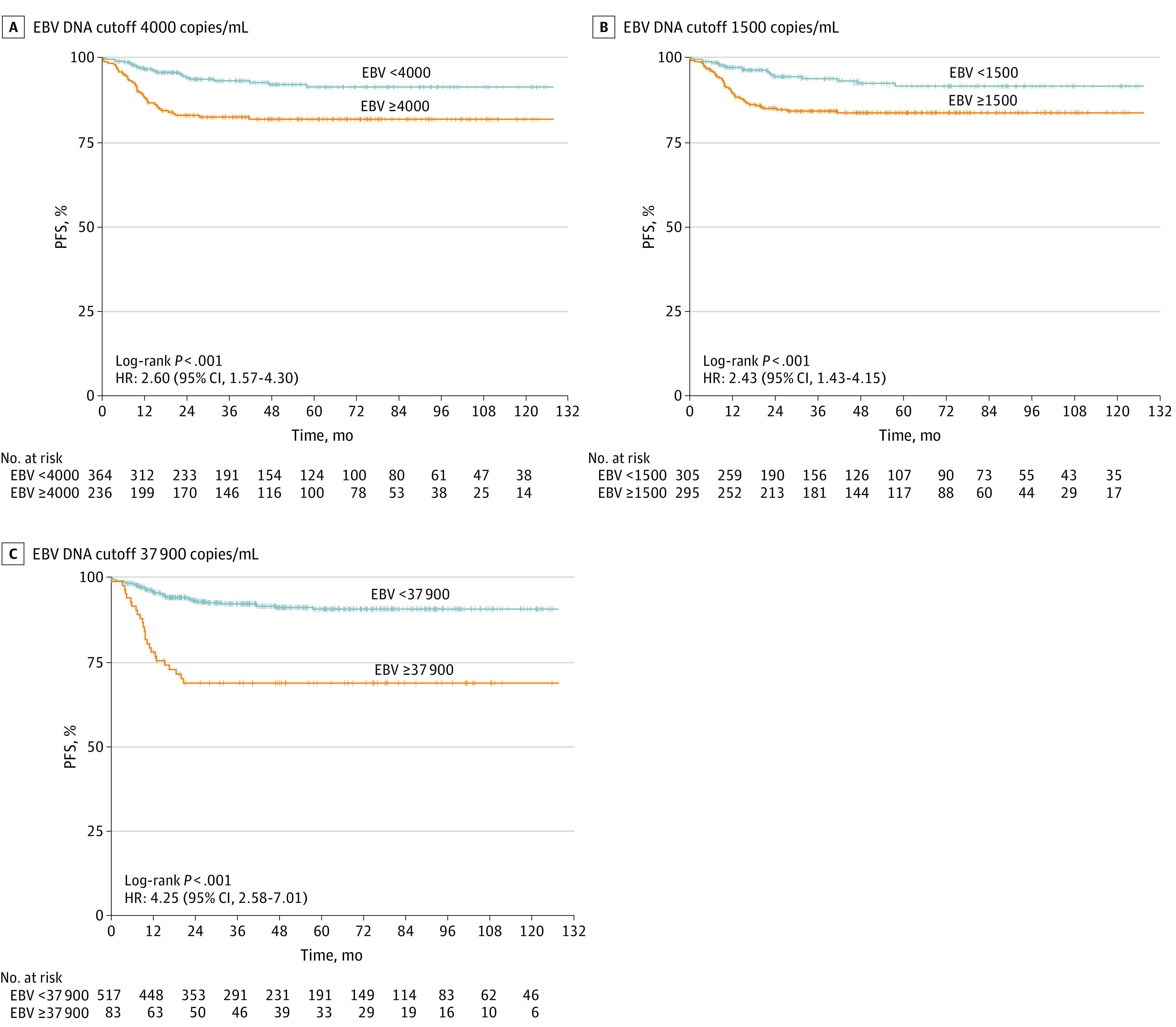
Kaplan-Meier Curves of Progression-Free Survival (PFS) by Epstein-Barr Virus (EBV) DNA Cutoff

In addition to the cutoff value of 1500 or 4000 copies/mL that was routinely used in adults,^13,14,21^ X-tile divided the data of pretreatment EBV DNA levels into high or low subsets with a cut point of 37 900 copies/mL in pediatric patients with NPC. Pediatric patients with NPC who were at high and low risk could be well distinguished in PFS with cutoff values of 4000, 1500, or 37 900 copies/mL, with HRs of 2.60 (95% CI, 1.57-4.30), 2.43 (95% CI, 1.43-4.15), and 4.25 (95% CI, 2.58-7.01), respectively (Figure 3).

## Discussion

To the best of our knowledge, this cohort study is the largest series to investigate associations between advances in techniques and treatment and survival rates in pediatric patients with NPC. In adult patients, locoregional recurrence-free survival rates improved dramatically in 2003 to 2012, while there was no obvious change in the distant metastasis–free survival rate over the calendar periods in 1990 to 2012.^18^ In pediatric patients with NPC in our study, the 5-year CIDM rate was approximately equivalent in 3 periods despite fluctuations, while the 5-year CILR rate decreased in the first 2 periods and then remained stable, suggesting that the development of technology may have been associated with satisfactory locoregional control while distant failure remained a key challenge.

The important role of MRI in image diagnosis was also suggested by the results of Liao et al.^7^ Compared with CT, MRI provides better resolution in assessing parapharyngeal spaces, bone infiltration, and intracranial diseases. The use of MRI has been associated with great changes in determining clinical staging, which directly affects treatment modality. In addition, MRI with contrast of the skull base to the clavicle is recommended as an image diagnosis technique in the National Comprehensive Cancer Network guidelines for individuals with NPC.^7,22^

Advanced RT techniques and RT in combination with chemotherapy were associated with better PFS in univariable analyses and were evident in subgroups considered. However, these positive associations were not present after multivariable adjustments, which did not completely coincide with results in adult patients with NPC.^18^ These findings may be explained as follows: Lai et al^16^ found that improved survival rates with IMRT compared with 2D-CRT were associated with increased local or regional control in early-stage disease, especially among patients with T1 disease. However, further improvement in local or regional control of patients with T3 or T4 disease has not been achieved, and distant control of NPC was still insufficient with IMRT.^16^ In our study, the number of patients in the early stage was too small to investigate advantages associated with local control of IMRT, with 20 patients with T1 disease (2.5%) owing to the lateness of diagnosis in pediatric patients with NPC. Moreover, in this special group of patients with NPC, accurate staging and plasma EBV DNA level were associated with prognosis, with significant differences in outcome for RT technique and chemotherapy in univariate analysis but not multivariate analysis. For treatment modality, a previous study^23^ reported that IC with CCRT was not associated with benefits in prognosis compared with CCRT. These findings suggest that early diagnosis and IMRT in combination with more effective treatment methods like targeted therapy or immunotherapy may be associated with further improvement of survival in patients with advanced-stage disease.^24^ In the GPOH (Gesellschaft für Pädiatrische Onkologie und Hämatologie) study group, excellent results were obtained with IC with CCRT followed by adjuvant interferon beta (IFN-β). Remarkably, the cumulative irradiation dosage was further reduced to 54 Gy in individuals with complete response after IC,^11^ suggesting the effectiveness and the possibility of improving quality of life associated with this treatment mode. Specifically, IC with cisplatin and 5-fluorouracil, CCRT, and considerable maintenance with IFN-β were recommended for selected pediatric patients with NPC at high risk and advanced stage.^12^ Additionally, 15 patients (3.2%) received RT alone in the IMRT era in the current study. Therefore, future efforts will be focused on comparing treatment modalities in more homogeneous patient groups.

In our study, CIDM rates were equivalent while CILR rates decreased. Improvement in locoregional control in 2003 to 2011 may be associated with the use of IMRT. Although IMRT became more popular in 2012 to 2020, the proportion of patients with stage IVa disease increased from 98 patients (46.2%) to 289 patients (60.7%); therefore, the association of IMRT with improved local control among early patients may not be reflected in our study.^16^ The CIDM rate increased during the 1989 to 2011 calendar period but decreased in 2012 to 2020. The proportion of patients in advanced stages (stages III-IVa) increased from 88.5% in 1989 to 2002 to 93.4% in 2003 to 2011. Meanwhile, the proportion of patients undergoing PET-CT that could detect and exclude patients with early metastases increased from 0 patients in 1989 to 2002 and 19 patients (9.0%) in 2003 to 2011 and 257 patients (54.0%) in 2012 to 2020.^21^ The CIDM and CILR rate in some subgroups of patients decreased and then rebounded or increased and then decreased. The main factor associated with this outcome was that the total number of patients in the exact subgroup or the number of patients with incidents was small, which was associated with fluctuations in survival rates.

Pretreatment plasma EBV DNA has been demonstrated as a reliable marker associated with progression in NPC, and a cutoff value of 1500 or 4000 copies/mL has been proposed mainly in adults in the endemic areas.^13,14,21^ To date, whether 1500 or 4000 copies/mL is the optimal cutoff value has not been investigated thoroughly, to our knowledge; neither has it been explored in pediatric patients with NPC. In our study, pretreatment plasma EBV DNA with a cutoff value of 4000, 1500, or 37 900 copies/mL was associated with high or low-risk of progression among pediatric patients with NPC well.

### Limitations

Our research also has some limitations. First, although patients were from a prospectively maintained database, there are inevitable biases owing to the retrospective and observational nature of the current study. Additionally, the results need to be verified in other centers. Second, missing values in EBV DNA were identified because this test was not available during 1989 to 2002, resulting in inevitable selection bias in multivariable regression analysis. In addition, the sources of bias were exacerbated when splitting patients into subgroups because of the Will Rogers phenomenon. Third, a longer follow-up time is needed for patients in 2012 to 2020. Fourth, standardized testing procedures for plasma EBV DNA have not yet been established globally, suggesting the need for standardized testing in the future.

## Conclusions

This study found that advanced techniques and treatment methods were associated with improved survival rates in pediatric patients with NPC. However, distant failure remained a key challenge, and future efforts should be focused on more effective therapeutic approaches to address this important clinical problem.

## Notes

Supplement. **eTable 1.** Univariate Analyses of Prognostic Factors by Outcome **eTable 2.** Univariate Cox Analysis of Variables With Missing Data in 5 Multiple Imputation Models **eTable 3. **Univariate Cox Analysis Comparing Association Between Variables and Progression-Free Survival in Complete Data or Models From Multiple Imputation **eTable 4.** Multivariable Analysis of Prognostic Factors Associated With Survival, 1980 to 2020 **eFigure 1.** Flowchart **eFigure 2.** 5-year Progression-Free Survival by Stage and Period With Mature Follow-up
