# Supplementary material for: Association of Treatment Advances With Survival Rates in Pediatric Patients With Nasopharyngeal Carcinoma in China, 1989-2020
Source: JAMA Netw Open. 2022 Mar 2;5(3):e220173. doi: 10.1001/jamanetworkopen.2022.0173 (PMC8892229; doi:10.1001/jamanetworkopen.2022.0173)
Supplement: Supplement. — eTable 1. Univariate Analyses of Prognostic Factors by Outcome eTable 2. Univariate Cox Analysis of Variables With Missing Data in 5 Multiple Imputation Models eTable 3. Univariate Cox Analysis Comparing Association Between Variables and Progression-Free Survival in Complete Data or Models From Multiple Imputation eTable 4. Multivariable Analysis of Prognostic Factors Associated With Survival, 1980 to 2020 eFigure 1. Flowchart eFigure 2. 5-year Progression-Free Survival by Stage and Period With Mature Follow-up [file jamanetwopen-e220173-s001.pdf]

Supplemental Online Content

Liang YJ, Liu LT, Li Y, et al. Association of treatment advances with survival rates in pediatric patients with nasopharyngeal carcinoma in China, 1989-2020. *JAMA Netw Open*. 2022;5(3):e220173. doi:10.1001/jamanetworkopen.2022.0173

eTable 1. Univariate Analyses of Prognostic Factors by Outcome

eTable 2. Univariate Cox Analysis of Variables With Missing Data in 5 Multiple Imputation Models

eTable 3. Univariate Cox Analysis Comparing Association Between Variables and Progression-Free Survival in Complete Data or Models From Multiple Imputation

eTable 4. Multivariable Analysis of Prognostic Factors Associated With Survival, 1980 to 2020

eFigure 1. Flowchart

eFigure 2. 5-year Progression-Free Survival by Stage and Period With Mature Follow-up

This supplemental material has been provided by the authors to give readers additional information about their work.

© 2022 Liang YJ et al. *JAMA Network Open.*

eTable 1. Univariate Analyses of Prognostic Factors by Outcome

**Survival**	**PFS**	**OS**	**CIDM**	**CILR**
Variables	HR (95%CI)	HR (95%CI)	HR (95%CI)	HR (95%CI)
PS	1.03 (0.65-1.64)	1.03 (0.59-1.79)	1.61 (0.98-2.64)	1.02 (0.48-2.15)
BMI	0.93 (0.89-0.98)	0.89 (0.83-0.96)	0.96 (0.9-1.01)	0.91 (0.83-1.01)
Sex	0.87 (0.62-1.24)	1.1 (0.70-1.71)	0.98 (0.63-1.53)	0.67 (0.39-1.15)
Age	1.09 (0.79-1.51)	1.08 (0.73-1.62)	1.07 (0.71-1.6)	1.1 (0.65-1.86)
Image technique				
CT	Reference	Reference	Reference	Reference
MRI	0.25 (0.17-0.38)	0.22 (0.14-0.36)	0.54 (0.33-0.90)	0.11 (0.05-0.25)
PET-CT + MRI	0.41 (0.27-0.62)	0.19 (0.10-0.37)	0.92 (0.56-1.50)	0.26 (0.13-0.53)
T stage	1.44 (1.04-2.00)	1.45 (0.97-2.16)	2.15 (1.43-3.22)	0.97 (0.56-1.68)
N stage				
0-1	Reference	Reference	Reference	Reference
2	0.85 (0.58-1.24)	0.92 (0.59-1.43)	0.81 (0.5-1.32)	1.32 (0.73-2.38)
3	1.46 (0.96-2.22)	1.23 (0.70-2.14)	1.83 (1.11-30)	0.99 (0.45-2.21)
Treatment				
RT alone	Reference	Reference	Reference	Reference
IC + RT	0.75 (0.45-1.27)	0.68 (0.36-1.28)	0.94 (0.45-1.99)	0.55 (0.25-1.22)
CCRT	0.55 (0.32-0.96)	0.53 (0.27-1.02)	0.82 (0.39-1.74)	0.60 (0.29-1.27)
IC + CCRT	0.59 (0.38-0.91)	0.6 (0.35-1.00)	1.13 (0.62-2.07)	0.27 (0.13-0.55)
CCRT/RT+AC	0.48 (0.25-0.91)	0.46 (0.19-1.14)	0.75 (0.33-1.74)	0.30 (0.1-0.88)
RT technique	0.42 (0.30-0.59)	0.34 (0.22-0.52)	0.81 (0.53-1.25)	0.23 (0.12-0.41)
Calendar periods				
1989-2002	Reference	Reference	Reference	Reference
2003-2011	0.45 (0.30-0.67)	0.37 (0.23-0.58)	1.59 (0.85-2.96)	0.11 (0.05-0.26)
2012-2020	0.27 (0.18-0.41)	0.14 (0.08-0.25)	0.88 (0.47-1.64)	0.13 (0.07-0.25)
VCA-IgA	1.16 (0.80-1.67)	1.28 (0.80-2.04)	1.07 (0.69-1.68)	1.17 (0.64-2.13)
EA-IgA	0.78 (0.56-1.10)	0.77 (0.51-1.16)	0.85 (0.55-1.32)	0.65 (0.37-1.15)

© 2022 Liang YJ et al. *JAMA Network Open.*

EBV DNA	2.6 (1.57-4.30)	2.50 (1.16-5.39)	2.47 (1.44-4.21)	1.03 (0.35-3.00)

Note: All HRs were adjusted for other covariates. p values were calculated with an adjusted Cox proportional hazard model in PFS and OS and with a sub-distribution hazard function model in CIDM and CILR.

HRs were calculated for PS (70-80 vs 90-100); Sex (Male vs Female); Age (>18 vs ≤18); T stage (T4 vs T1-3); N stage (N2, N3 vs N0-1); image technique (MRI vs CT); RT technique (IMRT/TOMO vs 2D-CRT/3D-CRT); treatment (RT+ chemotherapy vs RT alone), VCA-IgA (≥80 vs <80); EA-IgA (≥10 vs <10)and EBV DNA (≥4000 vs <4000 copies/ mL)

Abbreviations: NPC=nasopharyngeal carcinoma; EBV DNA=Epstein-Barr virus (EBV) DNA; IC=induction chemotherapy; AC=adjuvant chemotherapy; CT=computed tomography; RT= radiotherapy; CCRT=concurrent chemoradiotherapy; IMRT=intensity modulated radiation therapy; MRI=magnetic resonance imaging; IC=induction chemotherapy; PET=positron emission computer tomography; RT=radiation therapy; 2DRT= 2-dimensional radiation therapy; EA = early antigen; VCA = viral capsid antigen; IgA = immunoglobulin; WHO = World Health Organization; BMI = body mass index; PS= performance- status score (Karnofsky Performance Scale or Lansky Play-Performance Scale); PFS= progression-free survival; OS=overall survival; CIDM=cumulative incidence of distant metastasis; CILR=cumulative incidence of locoregional recurrence; HRs= hazard ratios

© 2022 Liang YJ et al. *JAMA Network Open.*

eTable 2. Univariate Cox Analysis of Variables With Missing Data in 5 Multiple Imputation Models

Multiple imputation comparing observed complete case data for progression-free survival

	**MI model 1**	**MI model 2**	**MI model 3**	**MI model 4**	**MI model 5**
**Variables**	HR (95%CI)	HR (95%CI)	HR (95%CI)	HR (95%CI)	HR (95%CI)
BMI	0.93 (0.89, 0.98)	0.92 (0.88, 0.97)	0.93 (0.88, 0.97)	0.93 (0.88, 0.98)	0.93 (0.89, 0.98)
VCA-IgA	1.16 (0.82, 1.65)	1.12 (0.79, 1.59)	1.17 (0.82, 1.67)	1.18 (0.83, 1.69)	1.25 (0.88, 1.80)
EA-IgA	0.76 (0.55, 1.05)	0.77 (0.56, 1.07)	0.85 (0.61, 1.17)	0.80 (0.58, 1.11)	0.75 (0.54, 1.04)
EBV DNA	2.00 (1.44, 2.78)	2.29 (1.64, 3.20)	2.28 (1.63, 3.19)	2.33 (1.68, 3.24)	2.33 (1.67, 3.25)

Abbreviations: EBV DNA=Epstein-Barr virus (EBV) DNA; EA = early antigen; VCA = viral capsid antigen; IgA = immunoglobulin; BMI = body mass index; MI= multiple imputation; HR= Hazard ratios

© 2022 Liang YJ et al. *JAMA Network Open.*

eTable 3. Univariate Cox Analysis Comparing Association Between Variables and Progression-Free Survival in Complete Data or Models From Multiple Imputation

	No. (%) of missing data	Complete case	Pool estimates from MI
**Variables**		**HR (95%CI)**	**HR (95%CI)**
BMI	62 (7.65%)	0.93 (0.89, 0.98)	0.93 (0.89, 0.98)
VCA-IgA	47 (5.80%)	1.16 (0.80, 1.67)	1.17 (0.82, 1.69)
EA-IgA	47 (5.80%)	0.78 (0.56, 1.10)	0.78 (0.56, 1.10)
EBV DNA	210 (25.9%)	2.60 (1.57, 4.30)	2.30 (1.54, 3.43)

Abbreviations: EBV DNA=Epstein-Barr virus (EBV) DNA; EA = early antigen; VCA = viral capsid antigen; IgA = immunoglobulin; BMI = body mass index; MI= multiple imputation; HR= Hazard ratios

© 2022 Liang YJ et al. *JAMA Network Open.*

eTable 4. Multivariable Analysis of Prognostic Factors Associated With Survival, 1980 to 2020

Factors are associated wtih progression-free survival, overall survival, distant metastasis-free survival, and locoregional recurrence-free survival of the 810 Childhood and Adolescent patients with NPC from 1989 to 2020

Survival	PFS	OS	CIDM	CILR
Variables	HR (95%CI)	HR (95%CI)	HR (95%CI)	HR (95%CI)
BMI	0.97 (0.92-1.02)	0.94 (0.88-1.01)	0.97 (0.92-1.03)	0.97 (0.89-1.06)
Image technique	0.41 (0.21-0.76)	0.33 (0.16-0.72)	0.54 (0.28-1.04)	0.47 (0.10-2.14)
T stage	1.81 (1.29-2.54)	1.99 (1.29-3.06)	2.45 (1.62-3.70)	1.39 (0.79-2.46)
N stage				
0-1	Reference		Reference	
2	1.09 (0.73-1.62)	1.18 (0.74-1.89)	0.98 (0.59-1.62)	1.59 (0.83-3.04)
3	2.36 (1.47-3.79)	2.28 (1.22-4.23)	2.54 (1.39-4.64)	1.69 (0.69-4.10)
Treatment	1.11 (0.68-1.80)	1.37 (0.80-2.36)	0.72 (0.36-1.43)	1.35 (0.66-2.74)
RT technique	0.99 (0.55-1.77)	1.41 (0.71-2.77)	1.00 (0.54-1.88)	0.82 (0.15-4.37)
Calendar periods				
1989-2002	Reference		Reference	
2003-2011	0.67 (0.37-1.22)	0.48 (0.23-0.97)	2.46 (1.14-5.30)	0.17 (0.04-0.72)
2012-2020	0.46 (0.20-1.08)	0.19 (0.06-0.54)	1.32 (0.48-3.62)	0.25 (0.03-2.32)
EBV DNA	1.52 (1.09-2.11)	1.50 (1.00-2.26)	1.59 (1.05-2.39)	1.02 (0.59-1.75)

Note: EBV DNA was not available (NA) in 1989-2002 since this test was carried out mainly after 2003, multiple imputation (MI) was also used to estimate the missing values.

All HRs were adjusted for other covariates. p values were calculated with an adjusted Cox proportional hazard model in PFS and OS and with a sub-distribution hazard function model in CIDM and CILR.

HRs were calculated for T stage (T4 vs T1-3); N stage (N2, N3 vs N0-1); image technique (MRI vs CT); RT technique (IMRT/TOMO vs 2D-CRT/3D-CRT); treatment (RT+ chemotherapy vs RT

© 2022 Liang YJ et al. *JAMA Network Open.*

alone), and EBV DNA (⩾4000 vs <4000 copies/ mL)

Abbreviations: EBV DNA=Epstein-Barr virus (EBV) DNA; RT=radiotherapy; BMI=body mass index; PFS=progression-free survival; OS=overall survival; CIDM=cumulative incidence of distant metastasis; CILR=cumulative incidence of locoregional recurrence; HRs=hazard ratios

© 2022 Liang YJ et al. *JAMA Network Open.*

eFigure 1. Flowchart

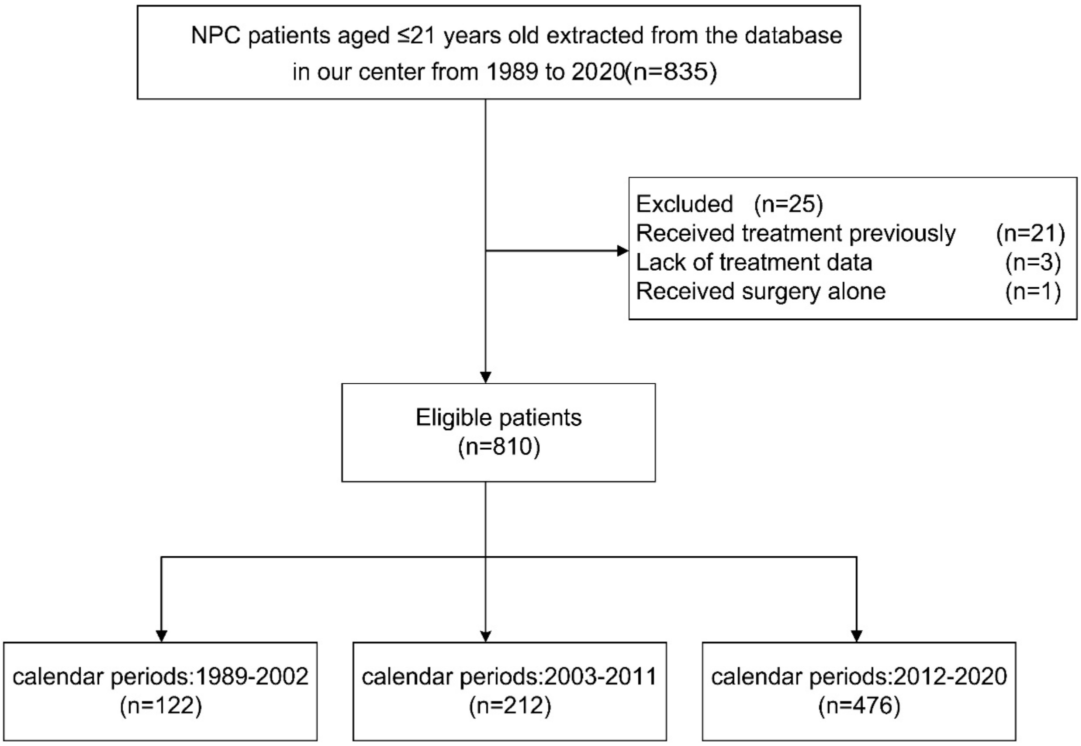

© 2022 Liang YJ et al. *JAMA Network Open.*

eFigure 2. 5-year Progression-Free Survival by Stage and Period With Mature Follow-up

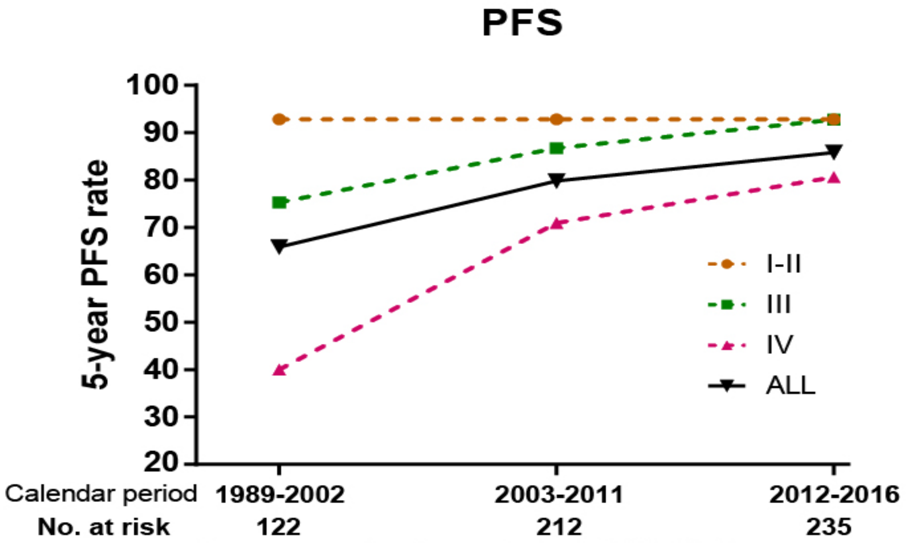

5-year progression-free survival rate (95% CI), %

UICC8th	1989-2002	2003-2011	2012-2016
I-II	92.9 (59.1,99.0)	92.9 (59.1,99.0)	92.9 (59.1,99.0)
III	75.3 (62.8,84.1)	86.8 (78.3,92.1)	92.7 (84.5,96.7)
IV	40.0 (24.6,55.0)	70.9 (60.7,78.9)	80.6 (72.6,86.5)
**ALL**	**65.9 (56.6,73.7)**	**79.8 (73.7,84.7)**	**85.8 (80.5,89.8)**

© 2022 Liang YJ et al. *JAMA Network Open.*
